# How do plants defend themselves against pathogens-Biochemical mechanisms and genetic interventions

**DOI:** 10.1007/s12298-022-01146-y

**Published:** 2022-03-07

**Authors:** Simardeep Kaur, Mahesh Kumar Samota, Manoj Choudhary, Mukesh Choudhary, Abhay K. Pandey, Anshu Sharma, Julie Thakur

**Affiliations:** 1 Division of Biochemistry, ICAR-Indian Agricultural Research Institute, New Delhi, India; 2 HCP Division, ICAR-CIPHET, Abohar, 152116 Punjab India; 3 ICAR-National Research Center for Integrated Pest Management, New Delhi, India; 4 Department of Plant Pathology, University of Florida, Gainesville, United States; 5 School of Agriculture and Environment, The University of Western Australia, Perth, Australia; 6 ICAR-Indian Institute of Maize Research, PAU Campus, Ludhiana, India; 7 Department of Mycology and Microbiology, Tea Research Association-North Bengal Regional R & D Center, Nagrakata, West Bengal 735225 India; 8 Department of FST, Dr. YS Parmar UHF Nauni, Solan, India; 9 Department of Botany, Bhaskaracharya College of Applied Sciences, University of Delhi, Delhi, India

**Keywords:** Induced resistance, Antioxidative components, Phytoalexin, Melatonin, PR proteins, Polyamines

## Abstract

In agro-ecosystem, plant pathogens hamper food quality, crop yield, and global food security. Manipulation of naturally occurring defense mechanisms in host plants is an effective and sustainable approach for plant disease management. Various natural compounds, ranging from cell wall components to metabolic enzymes have been reported to protect plants from infection by pathogens and hence provide specific resistance to hosts against pathogens, termed as induced resistance. It involves various biochemical components, that play an important role in molecular and cellular signaling events occurring either before (elicitation) or after pathogen infection. The induction of reactive oxygen species, activation of defensive machinery of plants comprising of enzymatic and non-enzymatic antioxidative components, secondary metabolites, pathogenesis-related protein expression (e.g. chitinases and glucanases), phytoalexin production, modification in cell wall composition, melatonin production, carotenoids accumulation, and altered activity of polyamines are major induced changes in host plants during pathogen infection. Hence, the altered concentration of biochemical components in host plants restricts disease development. Such biochemical or metabolic markers can be harnessed for the development of “pathogen-proof” plants. Effective utilization of the key metabolites-based metabolic markers can pave the path for candidate gene identification. This present review discusses the valuable information for understanding the biochemical response mechanism of plants to cope with pathogens and genomics-metabolomics-based sustainable development of pathogen proof cultivars along with knowledge gaps and future perspectives to enhance sustainable agricultural production.

## Introduction

The biotic stress constantly affects the agro-ecosystem which includes viruses, mycoplasmas, bacteria and fungi which directly alter the soil health and fertility and productivity of crops. Pathogens disturb the physiological and metabolic processes and pathways in plants resulting in loss of yield and quality in plants (Kumar and Verma 2018). Among all the processes, components of biochemical pathways play a vital part in safeguarding plants against pathogens. In general, when there is no stress, the plants exhibit optimal growth and development by using the available oxygen. However, during stress like pathogen attack, usage of oxygen results in the production of reactive oxygen species (ROS) in plant tissues (Singla et al. 2019), which in turn causes photo-oxidative damage to biomolecules and the internal cellular structures (Xie et al. 2016; Mittler 2017). The plants respond to such interaction of microbes by inducing a plethora of biochemical changes associated with stress signaling and thus activating their defense pathways. The induced defense mechanism includes various non-enzymatic components comprising phenolic compounds, flavonoids, lignins and enzymes for phenol metabolism like phenylalanine ammonia-lyase (PAL), polyphenol oxidase (PPO) and antioxidant enzymes like superoxide dismutase (SOD), catalase (CAT), peroxidases (POX) and glutathione reductase (GR) (Debona et al. 2012; Akter et al. 2015), accumulation of tannins and phytoalexins. Besides, changes in cell wall composition act as a passive structural fence against pathogen attack. Das and Roychoudhury (2014a, b) reported that carotenoids and lipophilic organic compounds also serve as a defense mechanism for the detoxification of several types of ROS. Another defensive compound such as phytoalexins is also produced in host plants as secondary metabolites by the hypersensitive response. During the pathogen infection, phytoalexins accumulate at the infection site and prevent the fungal growth and also of other pathogens *in-vivo* and thus, considered as an important plant-defensive compound against many necrotrophic and biotrophic pathogens (Bizuneh 2020). Apart from this, polyamines (PAs) also play a key function in the plant’s cellular metabolism and hence act as a protective barrier to pathogens by alteration of their activities (Hussain et al. 2011). Hence, the plants are well equipped with natural biochemical compounds to cope with the plant pathogens. Therefore, the present review is aimed to deliver a synthesis of the literature that discusses the function of various plant biochemical components in self-defense against pathogens. Furthermore, we also aimed to discuss the plant defense systems against pathogens through elaborating on the production of ROS, alteration in metabolites, antimicrobial compounds and their role in defense and modification of cell wall composition. Besides, the use of metabolic/biochemical markers for the development of pathogen-resistant cultivars has also been discussed.

## Plant disease resistance genes

During the plant-pathogen interaction, the plant releases several types of elicitors. These elicitors are recognized by plant resistance genes and trigger various biochemical and physiological changes in plants. Plant pathogens, pathogen-associated molecular patterns (PAMPs) are recognized by pattern recognition receptors (PRRs) and damage-associated molecular patterns (DAMPs) by wall-associated kinases (WAKs) within the cell membrane. Recognition and signaling cascade lead to pathogen triggered immunity (PTI). Other elicitors, generally called effectors are intercepted nucleotide-binding domains and leucine-rich repeats (NLRs) and this kind of recognition by R gene and defense response is called effector-triggered immunity (ETI) (Jones and Dangl 2006). PTI and ETI are interconnected and complement each other defense pathways instead of the earlier “Zig-Zag” way (Ngou et al*.* 2021). PRRs, WAKs, and NLRs mediated interconnected signaling cascades regulated Mitogen-activated protein kinases (MAPKs), ubiquitin, transcription factors (TFs), calcium, hormones, G-proteins expression in the plant (Gururani et al. 2012; Meng and Zhang 2013; Andersen et al., 2018). This leads to various defense responses that reduce pathogen spread; cell wall modification, closure of stomata, production of ROS, hypersensitive response, or the production of various anti-pathogen proteins and compounds (e.g., protease inhibitors, chitinases, defensins, and phytoalexins) (van Loon et al. 2006).

## Plant defense system against plant pathogens

In natural habitats, plant defense systems serve a pivotal role in safeguarding plants against pathogens and in nutrient mobilization (Miller et al. 2010). In addition to self-defense of plants, useful microbes and plant growth-promoting rhizobacteria also activate the defense mechanism via two different pathways, systemic acquired resistance (SAR) and induce systematic resistance (ISR). The ISR may be strengthened through beneficial microorganisms, whereas SAR implies an altered gene expression at molecular levels and is related to PR proteins. Nawrocka and Małolepsza (2013) reported that both ISR and SAR, have different gene expression and induction mechanisms that depend on the regulatory pathway. Under biotic stress, beneficial microbes stimulate SAR which includes an accumulation of PR protein and salicylic acid (SA), however, ISR depends on jasmonate and ethylene regulated pathways (Salas-Marina et al. 2011). The biochemical mechanism of self-defense in plants (induced by themselves) against pathogens has been covered under the following sections.

### Production of ROS and its role in protection mechanism

ROS production is significant to carry out a hypersensitive response for host defense. The balance between synthesis and removal of ROS is directly interrupted by biotic or abiotic stress (Mittler 2017). A common consequence in the cell under any stress is the production of ROS, viz., hydrogen peroxide (H_2_O_2_), superoxide anion radical (O_2_^•−^), singlet oxygen (^1^O_2_) and hydroxyl radical (^•^OH) that could lead to extreme oxidative loss to plant tissues. In higher plants, lower levels of ROS have been found to regulate differentiation, redox homeostasis, stress signaling and systemic responses, however, elevated levels of ROS harm cellular components through protein damage, lipid peroxidation and membrane destruction (Das and Roychoudhury 2014a, b). Thus, high ROS affects normal cellular functioning (Asthir et al. 2010). Under stress conditions, plants send signals to alter their metabolism for the synthesis/activation of defensive genes in affected plant parts (Gill et al. 2019).

During stressful conditions, the ROS production rate dramatically increases in plants’ mitochondria, chloroplast, endoplasmic reticulum, apoplast, peroxisomes, plasma membrane and cellular walls (Sharma et al. 2012; Mittler 2017). ROS affects the lipids, proteins as well as DNA in plant cells. During stressful conditions, lipid peroxidation increases significantly through the formation of lipid radicals (Meo et al. 2016). ROS also causes oxidation and modifications of the proteins directly or indirectly. Direct changes include nitrosylation, carboxylation, the formation of disulfide bonds and glutathionylation. Interaction of lipid peroxidation products with protein may also lead to indirect changes. Various amino acids like proline, lysine, threonine, arginine, methionine and cysteine are highly susceptible to ROS attack (Petrov et al. 2015). ROS also causes damage to DNA at multiple sites that include changes in nitrogenous bases, breakage of DNA strands, oxidation of deoxyribose sugar, etc. If the cross-linking between DNA and protein is not repaired in time it is very harmful and deadly to the plant (Popracet al. 2017). ROS détoxifications are carried out when plant cells, enzymes and redox metabolites function synergistically to protect themselves from adverse effects. Oxidative stress tolerance is an integrated mechanism associated with the changes in antioxidative/defensive enzymes, free radical scavenging activities, non-enzymatic antioxidants, osmolytes and signaling molecules (Caverzan et al. 2016). The induced defense is facilitated via defensive enzymes i.e., CAT, SOD, POX, PAL, PPO, ascorbate peroxidase (APX) and tyrosine ammonia-lyase (TAL) along with secondary metabolites such as phenols and condensed tannins and also through the utilization of H_2_O_2_ and malondialdehyde (MDA) (Bhaduri and Fulekar 2012). During various environmental stresses or pathogenic attacks, the first line of defense is formed by SOD against ROS-induced loss in barley (Torun et al. 2019).

**Fig. 1:**
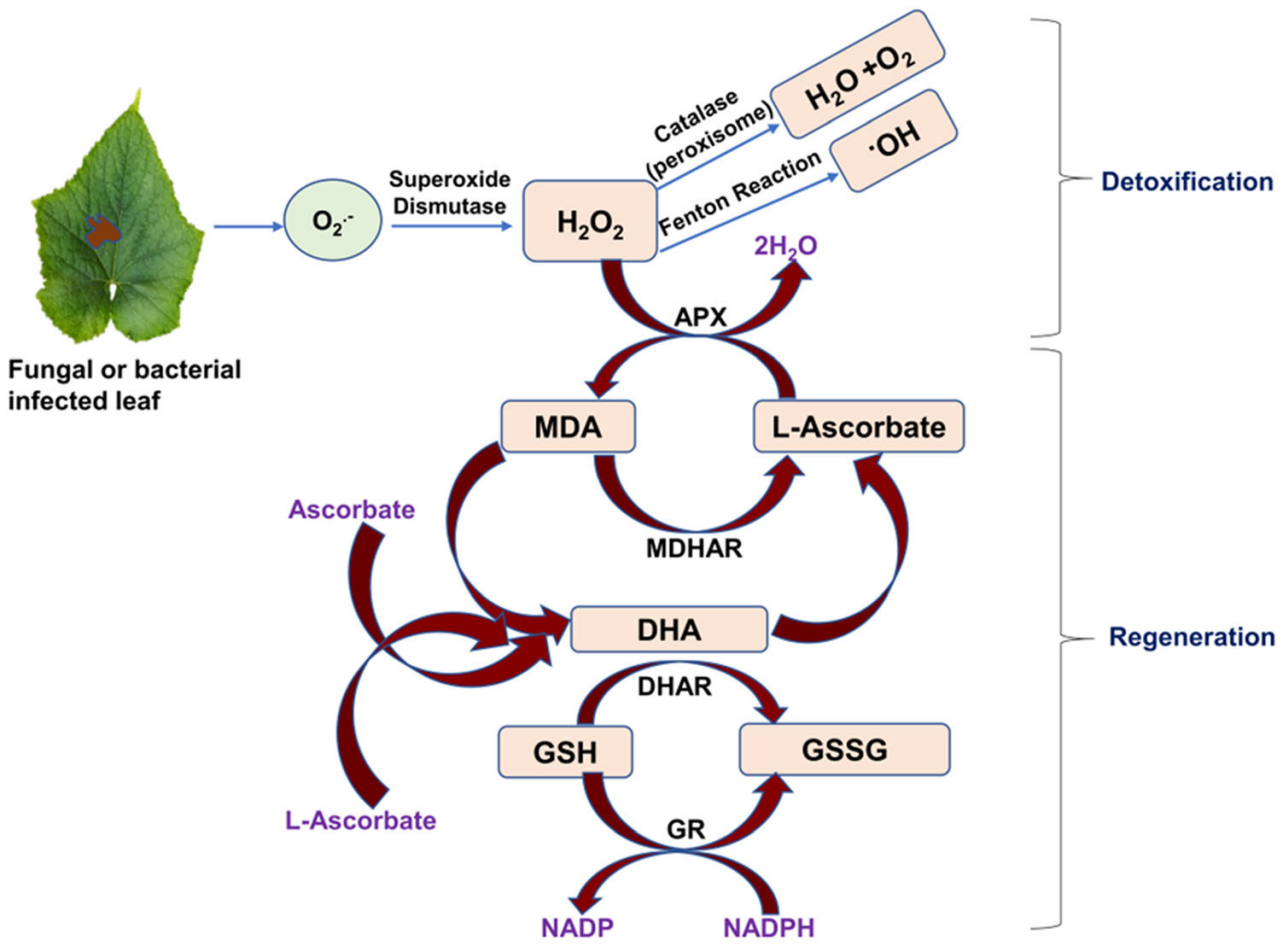
Antioxidant defense system in plants to detoxify the reactive oxygen radicals generated during the stress condition. The induced defense is facilitated via defensive enzymes such as Peroxidases, Catalase, Superoxide Dismutase (SOD) and Ascorbate peroxidase (APX).SOD catalyzes the dismutation of O_2_^–^ to H_2_O_2_, catalase (CAT) dismutases H_2_O_2_ to oxygen and water, and ascorbate peroxidase (APX) reduces H_2_O_2_ to water by utilizing ascorbate (ASC) as the specific electron donor. This antioxidant defense system is considered the main enzymatic system for protecting cells against oxidative damage

Major antioxidative enzymes that perform a significant role in plant-defense mechanism are discussed in Table 1 and Fig. 1.

**Table 1:** Description of defensive enzymatic machinery of plants against pathogens

Name of enzyme	Description	Isoforms/types	Function	Reaction catalyzed	References
Superoxide dismutase (SOD)	Family of metalloenzymes present in all organisms. During various environmental stresses or pathogenic attacks, SOD forms the first line of defense against ROS induced damages	1. Mn-SOD (localized in mitochondria) 2. Fe-SOD (present in chloroplasts) 3. Cu/Zn-SOD (residing in cytosol, peroxisomes, and chloroplasts) 4. Increased activity of SOD provides resistance against *Alternaria solani* in tomato	Removal of O^•^_2_^−^via dismutation it into O_2_ and H_2_O_2_	$$\begin{gathered} {\text{O}}_{{2}}^{ \cdot - } + {\text{ O}}_{{2}}^{ \cdot - } + {\text{ 2H}}^{ + } \hfill \\ \Downarrow \hfill \\ {\text{2H}}_{{2}} {\text{O}}_{{2}} + {\text{ O}}_{{2}} \hfill \\ \end{gathered}$$	Torun et al*.* (2019) Gulzar et al. (2021)
Catalase (CAT)	Tetrameric heme-containing enzyme and has high affinity for H_2_O_2_ and has a completely high turnover rate (6 × 10^6^molecules of H_2_O_2_ to H_2_O and O_2_ in one minute)	1. CAT1 which is expressed in pollen and seeds (residing in peroxisomes and cytosol) 2. CAT2 in photosynthetic tissues (present in peroxisomes and cytosol) 3. CAT3 which is present in leaves and vascular tissues (localized within the mitochondria) 4. CAT activity increases in leaves of barley and provides resistance against *Bipolaris sorokiniana* *5.* Induction of CAT activity provide resistance against S*clerotium rolfsii* in chickpea	Dismutation of H_2_O_2_ into H_2_O and O_2_	$$\begin{gathered} {\text{H}}_{{2}} {\text{O}}_{{2}} \hfill \\ \Downarrow \hfill \\ {\text{H}}_{{2}} {\text{O }} + \, \left( {^{{1}} /{2}} \right){\text{ O}}_{{2}} \hfill \\ \end{gathered}$$	Das and Roychoudhury (2014a, b) Sandalio et al. (2021) Bhaduri and Fulekar (2012) Kaur et al. (2021) Sahni and prasad (2020)
Ascorbate peroxidase (APX)	Class I superfamily of heme peroxidases	Exists in diverse isoforms viz*.* cytosolic, stromal, thylakoidal, mitochondrial and peroxisomal Up-regulated activity of peroxidase in rice provides resistance against *Xanthomonas oryzae* Upregulated activity of peroxidase shows resistance against powdery mildew disease in cucumber	It utilizes ascorbate as H-donor to breakdown H_2_O_2_ and releases water and monodehydroascorbate (MDHA) (Fig. 1)	$$\begin{gathered} {\text{H}}_{{2}} {\text{O}}_{{2}} + {\text{ AA}} \hfill \\ \Downarrow \hfill \\ {\text{2H}}_{{2}} {\text{O }} + {\text{ DHA}} \hfill \\ \end{gathered}$$	Chiang et al., (2015) Caverzan et al. (2016) Kalaivani et al. (2021) Jogaiah et al. (2020)
Glutathione reductase (GR)	GR is a flavoprotein and an oxidoreductase located in both eukaryotes and prokaryotes	Mainly, it is present in chloroplasts with little amounts present inside the mitochondria and cytosol Increased activity of GR provide resistance against *Alternaria solani* in tomato	It catalyses reduction of GSSG in NADPH dependent manner and thus is critical in maintaining GSH pool	$$\begin{gathered} {\text{GSSG }} + {\text{ NADPH}} \hfill \\ \Downarrow \hfill \\ {\text{2GSH }} + {\text{ NADP}} \hfill \\ \end{gathered}$$	Bela et al., (2018) Dey et al. (2016) Gulzar et al. (2021)

### Structural modifications in host plants

The plant cell wall is the primary target site of infection by the pathogen. Over a million years of co-evolution, plants have evolved a multilayered defense mechanism against microbes, of which the cell wall is a vital component. Microbes establish a pathogenic relationship with host plants by avoiding the plant cell wall which requires suitable host recognition tactics followed by the appropriate infection structures and/or chemical exudates formation (Turra et al. 2015). The microbes that fail to evolve suitable tactics to bypass the cell wall of a host plant remain as non-pathogens or non-adapted pathogens. In the case of pathogenic microbes, with the ability to overcome preformed barriers, the host plant uses the cell wall as a defense barrier. Some of the mechanisms include:

#### Release of elicitors

Boller and Felix (2009) reported the release of oligosaccharide elicitors during infection from the cell wall of a host plant DAMPs or of a pathogen PAMPs as a part of the process of degradation. Plants recognize these elicitors with a help of immune receptors present on the plasma membrane which eventually trigger signaling cascades to activate various DAMP or PAMP-triggered immunity defense mechanisms, these are also known as Defense Triggered Immunity (DTI) or PTI, respectively (Jones and Dangl 2006). One of the common defense responses among DTI or PTI is the cell wall reinforcement to develop more resistance to physical pressure and/or hydrolytic enzyme produced by the pathogens (Malinovsky et al. 2014). Moerschbacher and Mendgen (2012) reported that the reinforcement of the cell wall process may take place by various means depending upon the type of interaction with elicitors such as cross-linking and rearrangement of pre-existing cell wall components, the inclusion of cross-linked polymerized materials to the existing cell wall and local cell wall components deposition at the site of infection. For example, the elicitors induce thickening of outer layer parenchyma cells and produce amorphous, fibrillar material to trap the bacteria (Keane 2012). In contrast to bacteria, various defensive substances like callose, hydroxyproline amino acid-rich glycoproteins (such as an extension), phenolic compounds (also lignin as well as suberin) and mineral elements (e.g., calcium and silicon) are produced and deposited into the cell wall to safeguard against fungus attack (Deepak et al. 2010).

#### Deposition of papillae

**Fig. 2:**
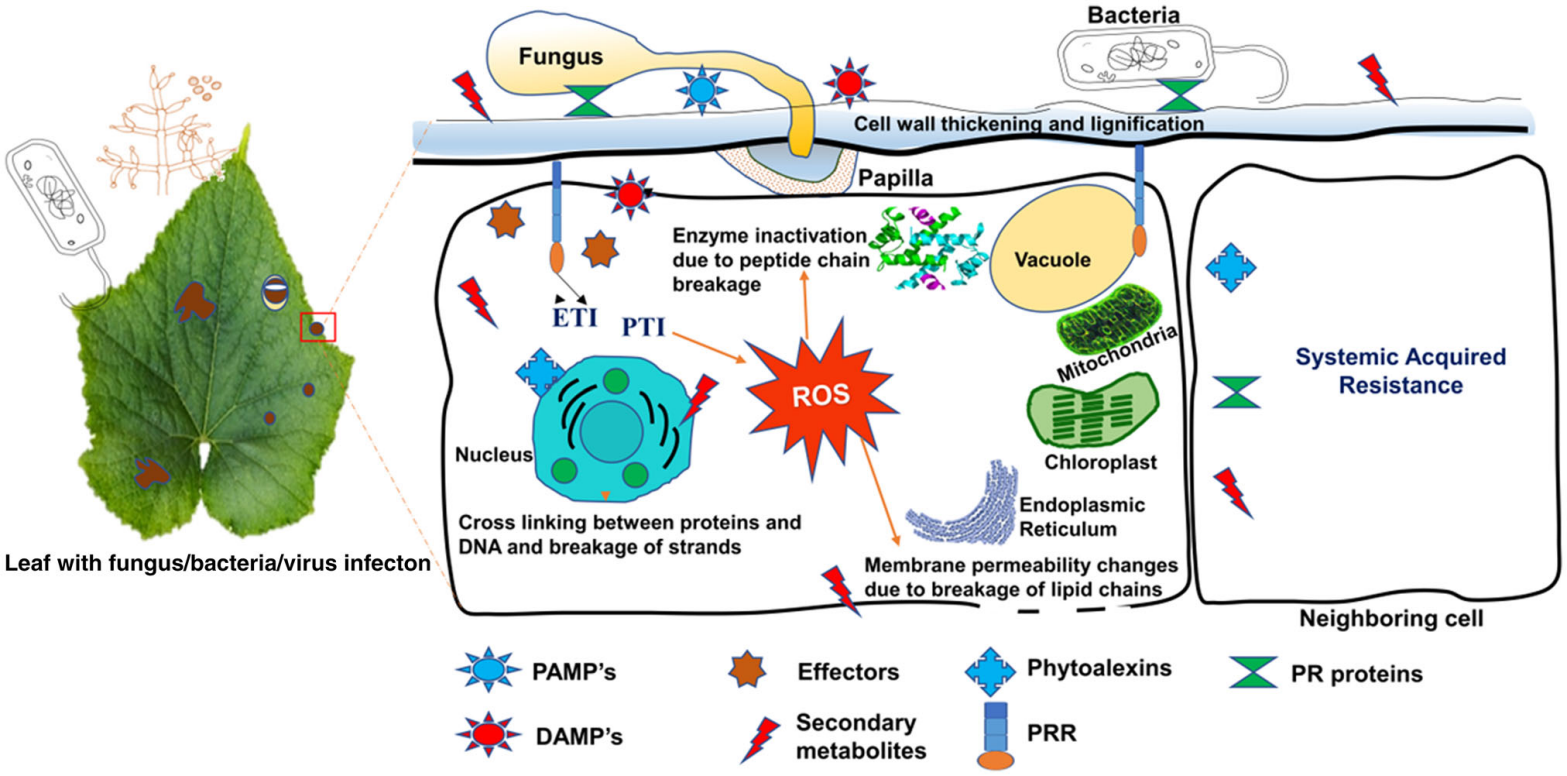
Schematic representation of biochemical defense response by plant cell in response to plant pathogen (fungus, bacteria, etc.). Fungal germ tube growth is restricted by thickening of cell walls and formation of papilla. Biochemical defense response is triggered by PAMP-triggered immunity (PTI) and effector-triggered immunity (ETI) by the production of reactive oxygen species (ROS), phytoalexins, pathogenesis related (PR) protein and secondary metabolites like phenylalanine ammonia lyase, polyphenol oxidase, flavonoids etc. PAMPs and DAMPs initiate the PRR mediated immune response. Various secondary metabolites also act as signal molecules and intermediates for systemic acquired resistance (SAR) against pathogens in plants

The local depository material of the cell wall is called papillae, which is formed between plant membranes and inside the plant cell wall. Papillae composition varies between plant species but phenolics, ROS, cell wall proteins and polymers are most commonly present among species. Among cell wall polymers (1, 3) -*β*- glucan callose is plentiful and invasive (Voigt 2014). For instance, Chowdhury et al. (2014) utilized microarray expression profiling to reveal the involvement of major polysaccharides, such as callose, arabinoxylan and cellulose in barley papillae produced in response to the pathogen- *Blumeria graminis* f. sp. *Hordei* (*Bgh*). They found that the effective papillae that prevent Bgh’s penetration contain more polysaccharides than ineffective papillae. The inner core of papillae is made up of callose and arabinoxylan and the outer layer consists of arabinoxylan and cellulose. The established interconnection between arabinoxylan and cellulose to penetration resistance offers new avenues for further refinement of the Composition of papillae and the development of cultivars/plants with better resistance against diseases. Recently, Li et al. (2018) showed the promising function of multi-vesicular bodies (specialized late formed endosomes) in papillae formation in response to bacteria and fungus (Li et al. 2018). Figure 2 explained the biochemical defense response by plant cells in response to a plant pathogen.

### Metabolic alteration and synthesis of antimicrobial compounds

Many plant species produce a wide variety of toxic secondary metabolites for pathogens. The functions of secondary metabolites in plant defense include deterrence, toxicity and as a precursor to the physical defense system. The metabolic alteration in hosts consists of the synthesis of many antimicrobial proteins, enzymes and metabolites. All these host responses provide enough strength and rigidity to decrease the wounds caused by pathogens. Some important plant metabolites, enzymes and proteins are discussed here under the following section.

#### Production of melatonin and its ecological roles against pathogens

Melatonin is derived from serotonin that acts as an effective biocide against pathogens e.g., bacterial and fungal. The low dose of melatonin confers antimicrobial resistance against gram-positive and negative pathogenic bacteria (Tekbas et al. 2008). Melatonin confers antimicrobial activity by upregulating the pathogen-related, SA and ethylene signaling-associated gene and reducing the plant’s susceptibility. Melatonin has also been found to be effective in decreasing the rate of infection after entry of the pathogens in plants (Arnao and Hernandez-Ruiz 2018), for example, the melatonin application also reduced the number of *Pseudomonas syringae* (the virulent bacterial pathogen) in infected leaves of *Arabidopsis thaliana* and also revealed the involvement of melatonin, especially serotonin (Fujiwara et al. 2010) and N-acetyl serotonin (Lee et al. 2014) in activating defense signal molecules (elicitor) that triggers the expression of numerous defense-related genes against *P. syringae* in Arabidopsis and tobacco (Lee et al. 2015). Furthermore, these findings were validated using diverse *Arabidopsis* mutants and hence revealing the involvement of melatonin action in the upstream of defense genes signaling pathway to biosynthesize various phytohormones i.e., salicylic acid, jasmonic acid and ethylene (Zhu et al. 2015) that together bring out disease resistance in a well-coordinated manner. Hence, the initial phase of plant-pathogen interaction results in enhanced ROS production (oxidative burst), which the plant takes care of by enhanced endogenous melatonin production (Lee et al. 2014). There are some interesting examples available, where fungi and bacteria live in plants in a mutualistic endophytic relationship and possess a higher level of melatonin than those plants where such mutualism does not occur (Jiao et al. 2016). Hence, this indicates an interesting area to examine the functions of melatonin levels in mutualistic relationships between plants and fungi to tackle the attack/infection against harmful microbes.

#### Elicitation of phytoalexins

Phytoalexins are de novo synthesized antimicrobial compounds by plants, as part of the action to defend against invading pathogens. Phytoalexins provide disease—resistance against pathogens but the mode of action varies depending on the types of host–pathogen interaction. The majority of phytoalexins are toxic and repress the growth of pathogenic fungi, nematodes and bacteria. Accumulation of phytoalexin is controlled by the relevant biosynthetic enzymes that are induced by biotic and abiotic stress-generated elicitors. The enhanced expression of biosynthetic enzyme coding genes increases the levels of phytoalexins. The response of the elicitors to induce the phytoalexin synthesis quickly (within minutes) stimulates de novo transcription of the corresponding genes. For example, Glyceollin (pathogen elicited phytoalexins) regulates resistance against *Phytophthora sojae* in soybean (Jahan et al. 2020. Like the localized cellular compartmentalization of phytoalexins during pathogen interaction, induction of the phytoalexin biosynthetic enzyme genes is also localized to the cells neighbouring the infection site. For example, Cocoa resistance to *V. alboatrum* has been linked with the localized phytoalexins production in the vicinity of the vessel (Cooper et al. 1996; Laouane et al. 2011). This helps to produce phytoalexins at higher rates and hence quickly controlling the pathogen proliferation in plants. The general function of the phytoalexin involves puncturing the cell wall of pathogens, disrupting their metabolism and reproductive functions and hence arresting the growth and development of invaded pathogens.

**Fig. 3:**
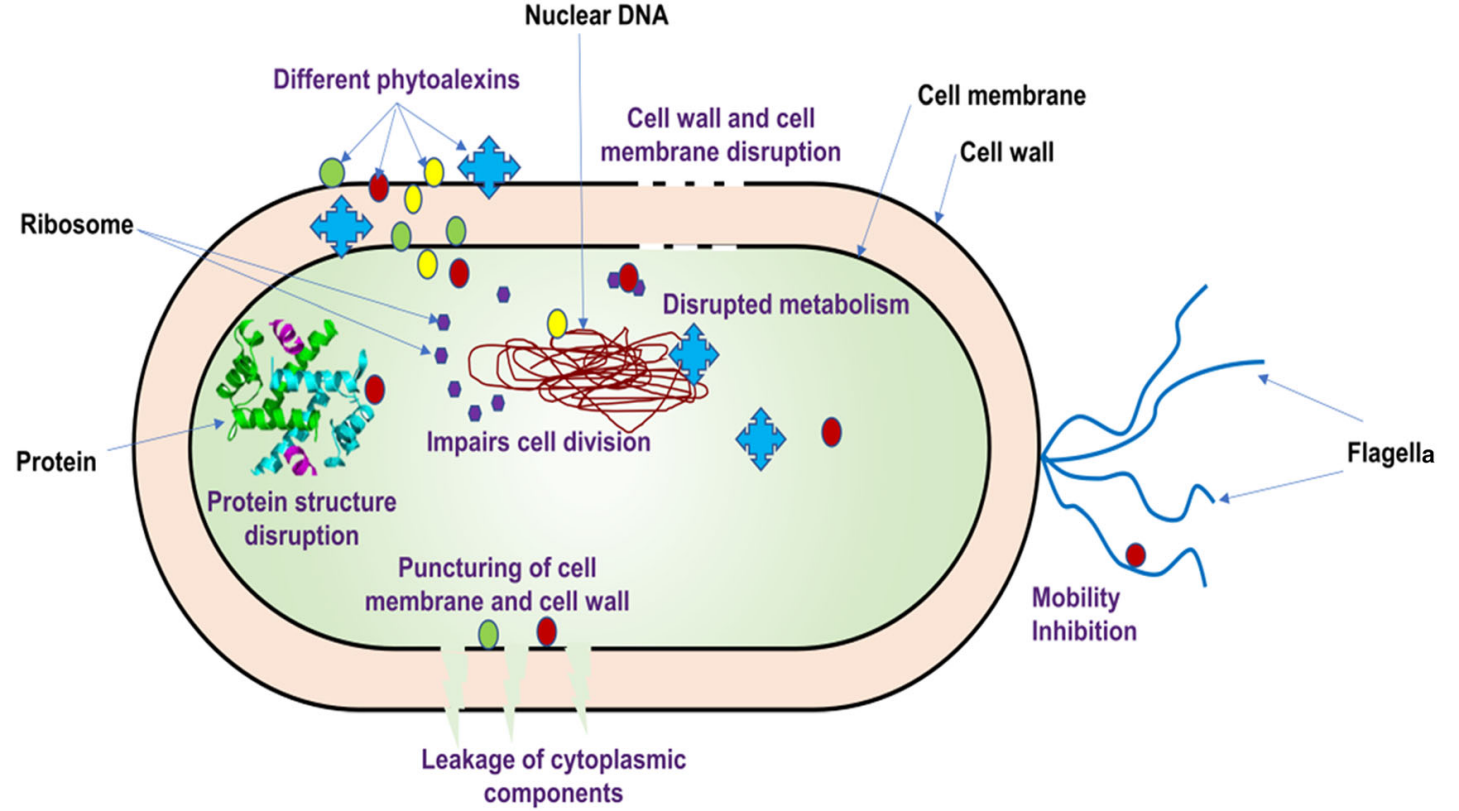
Diagrammatic representation of mechanisms of phytoalexins against bacteria. Phytoalexins act in many ways, with each phytoalexin having a specific mode of action. It can either directly affect the cell via membrane disruption and cell metabolites or indirectly by movement or cell multiplication

To date, over 300 chemicals having phytoalexin-like characteristics have been identified belonging to over 30 plant families. The phytoalexins are family-specific. For example, sulfur-containing phytoalexins (like brassin) are most common among all Brassicaceae, while the Poaceae family possesses different phytoalexins like oryzalexins, zealexins, kauralexins, sakuranetin and phenyl amides (Arruda et al. 2016). Similarly, phenylpropanoid-related compounds, steroid glycoalkaloids sesquiterpenoids and coumarins are found in Solanaceae; and isoflavones, coumestans, phaseollin, stilbenes/resveratrol are restricted to the family Leguminosae. The list of phytoalexins identified against different diseases in various crops is presented in Table 2 and the mechanism of phytoalexins against bacteria is represented in Fig. 3. Chemically, phytoalexins are different, however, the majority of phytoalexins are the product of the Shikimic acid pathway through which the majority of the secondary metabolites are derived such as anthocyanins, flavonoids and lignin. The key enzymes such as PPO, PAL, 4-coumarate-CoA ligase (4CL), cinnamate 4-hydroxylase (C4H) and chalcone synthase (CHS) involved in this pathway serve a significant part in defense- mechanisms.

**Table 2:** List of the different Phytoalexins identified in plants against various pathogens

Plants	Pathogens or elicitors	Biosynthesis pathways, signaling components and other defense responses	Phytoalexins	References
Alfa-alfa	*Fusarium oxysporum* f. sp. *medicaginis*	Flavonoid biosynthesis	Medicarpin and 7,4′-dihydroxyflavone	Gill et al. (2018)
Pea	*Nectriahaemato cocca* and *Mycosphaerella pinodes*	Pisatin biosynthesis, Pisatin tolerance	Pisatin	Coleman et al. (2011a, b)
Soybean	*Fusarium solani*	Phenylpropanoid pathway	Glyceollins	Abdul, M and Al-Muwayhi (2020)
	*Colletotricum truncatum*	Fatty acid synthesis pathway	Octanoic Acid	Nose et al. (2022)
Tobacco	*Botrytis cinerea* and *Phytophthora nicotianae*	Superoxide release, HR cell death	Scopoletin and capsidiol	El Oirdi et al. (2009)
	*A. tenuissi*	Adverse effects on mycelial growth	Biphenyl	Song et al. (2021)
Grape	*Agrobacterium* rhizogenes	Tyrosine phosphorylation, cell death	Resveratrol	Kiselev et al. (2007)
Maize	*Rhizopus microsporus*, *Colletotrichum graminicola*, *Fusarium graminearum*, *Cochliobolus heterostrophus* and *Aspergillusflavus*	Kauralexin synthesis and jasmonic acid-ethylene synergy	Kauralexins and zealexins	Schmelz et al. (2011)
	*Fusarium graminearum and Fusarium verticillioides*	Flavonoid Biosynthesis	Xilonenin	Forster et al. (2022)
Oat	*Puccinia coronata*	Avenanthramide biosynthesis	Avenanthramides	Yang et al. (2004)
Rice	*Magnaporthe oryzae*	Phytocassanes, momilactones and oryzalexin synthesis, and HR-associated phytoalexin biosynthesis	Momilactone A and momilactone B, phytocassane A, phytocassane E and sakuranein	Hasegawa et al. (2010); Ahuja et al. (2012)
		Diterpenoids	*Ent*-10-oxodepressin	Liang et al. (2021a, b)
		Flavonoids biosynthesis	Tangeretin	Liang et al. (2021a, b)
Sorghum	*Colletotrichum sublineolum* and *Cochliobolus heterostrophus*	Flavone biosynthesis from flavanones, H_2_O_2_ accumulation, papilla formation, callose deposition, HRGP cross-linking, cell death	Luteolin, apigenin and 3-deoxyanthocyanidins	Liu et al. (2010)
	*Fusarium*	Anthocyanins synthesis pathway	3-deoxyanthocynidin	Nida et al. (2021)
Sugarcane	*Colletotrichum falcatum*	Phenyl propanoid biosynthesis pathway	3-deoxy anthocyanidin	Nandakumar et al. (2021)
Barely	*B. sorokiniana* and *Fusarium graminearum*	Chalcone synthesis	Methoxylchalcones	Ube et al. (2021)
Lettuce	*Rhizoctonia solani* and *Olpidium virulentus*	Shikimate pathway	Benzoic acid and lettucenin A	Windisch et al. (2021)

#### Phenylalanine ammonia lyase (PAL) role in plant resistance

PAL, consisting of tetramer with each subunit of 77–83 kDa, is an extensively studied enzyme (Jendresen et al. 2015) as its the chief enzyme in the metabolism of phenylpropanoid and aids in synthesizing several secondary metabolites which include phenols (coumarins, flavonoids, lignins), phenolic derivatives and lignin (Bhattacharjee et al. 2017). The activity of PAL increases with the infection in several pathosystems (You et al. 2020). PAL activity in the leaves of resistant cultivars of barley genotypes increased against infection spot blotch pathogen *B. sorokiniana* (Kaur et al. 2021). Resistant reaction mainly occurs due to the defense of the cell walls by lignin intensification and the cell wall-bound phenolic compounds accumulation (Jun et al. 2018). In transgenic tobacco, reduction in lesion size and the number has also been witnessed in plants with PAL over-expression after getting infected with *P. syringae*. Similarly, bread wheat exhibited PAL gene AevPAL1 confers resistance to cereal cyst nematode by affecting the synthesis of salicylic acid and downstream secondary metabolites (Zhang et al. 2021)*.* These studies indicate that PAL over-expression in plants provides disease resistance.

#### Role of polyphenol oxidase (PPO) in plant resistance

PPO has three domains which include: i) an N-terminal plastid transit peptide ii) a highly conserved type-3 copper center and iii) a C-terminal part. The PPO catalyzes the oxidation of monophenols and o-diphenols to o-quinones and is broadly scattered amongst bacteria, fungi, plants and animals (Boeckx et al. 2015). Direct effect of PPO on photosynthesis in walnut (*Juglans regia*) and also as an oxygen buffer or water-water cycle to facilitate reactive oxygen scavenging (Araji et al. 2014). For example, in different pearl millet cultivars, the level of PPO was positively correlated with the incidence of downy mildew resistance (Raj et al. 2006). In another investigation, potato plants resistant to bacterial wilt exhibited relatively enhanced expression of PPO after infection with *Ralstonia solanacearum* than the susceptible plants (El-Argawy and Adss 2016). In tomato plants, the over-expressed PPO exhibited increased resistance to *P*. *syringae*. Khodadadi et al. (2020)reported transgenic tobacco lines reduced the severity of disease symptoms with a reduced population of bacteria.

#### Flavonoids

Knowingly, higher plants are rich in flavonoids that occur within the leaves and floral organs. Based on their chemical structure, they are categorized under four groups, namely, flavonols, flavones, isoflavones and anthocyanins. Flavonoids perform important roles in providing protection from pathogens as well as in pigmentation to flowers and other plant parts (Fini et al. 2011). In plants, flavonoids are regarded as one of the secondary ROS scavenging systems experiencing destruction to the photosynthetic pigments because of extra excitation power. Agati et al. (2012) reported that they are important in scavenging ^1^O_2_ and mitigating the destruction that happened to the outer envelope of the chloroplast membrane. For example, antibacterial flavonoids selectively target bacterial cells, inhibit virulence factors and reduce biofilm formation by interfering with quorum sensing (Gorniak et al. 2019). A study was carried out on the role of flavonoids on cotton wilt resistance. Metabolomics and transcriptomic analysis displayed flavonoids enrichment in leaves because of the upregulation of flavonoid biosynthesis genes. Also, in the red cotton cultivar, the fungal-pathogen invasion activity of *Verticillium dahliae* was suppressed because of the enhanced levels of flavonoids (Long et al. 2019). Identified Bayogenin 3-O-cellobioside (Norvienyeku et al. 2020), probenazole-inducible protein 1 (PBZ1), and phenylpropanoid accumulated (Ma et al. 2020) by metabolomics study of rice, are correlated with blast resistance. Metabolomics showed the accumulation of flavonoid compounds and plants coping with *C. gloeosporioides* by biosynthesis of flavonoid compounds providing potential targets for resistance breeding (Jiang et al. 2021).

#### Lignin deposition

Plants tend to deposit the lignin and callose in the cell wall to encounter the attack from pathogens. Enzymes such as CAD, PAL and POX are involved in the biosynthesis of lignin. Enhanced activity of these enzymes during pathogen infection indicates their substantial role in plant defense (Scott-Craig et al. 1995). Hence, one of the common responses of plant defense is enhanced lignification (Vance et al. 1980). For instance, wheat varieties that were infected with spot blotch-pathogen exhibited a better lignification response (Eisa et al. 2013). The lipid peroxidation leads to MDA production in response to stress conditions, therefore, MDA content is a lipid peroxidation indicator (Malencic et al. 2004) and used as a marker for cellular damage caused due to stress. Phenolic compounds can also contribute to strengthening host cell components with the aid of lignin and suberin biosynthesis providing act as a physical barrier to pathogens. For example, in olive plantations, “Olive Quick Decline Syndrome” tolerant cultivar has been shown to decrease in disease progression through lignification of cell wall against *X. fastidiosa* (Sabella et al. 2018). Similarly, in potatoes, leaf xylem tissue lignifications (induced by the biocontrol fungus *Trichoderma viride)* have been found to decrease the severeness of late blight disease (Purwantisari et al. 2020). The various biochemical metabolites and cell wall components that have major contributions to disease resistance in different crops are presented in Table 3.

**Table 3:** List of studies of various biochemical metabolites and cell wall components for providing disease resistance in different crops

S. No	Crop	Pathogen	Response	References
1	Barley	*Puccinia striiformis* f. sp. Hordei (causing stripe rust in barley)	Leaves of the resistant cultivar (RD 2901) showed an increase in activities of NADPH oxidase, catalase, peroxidase, and enzymes of ascorbate–glutathione pathway at the seedling stage	Singla et al. (2019)
2	Chickpea	*Helicoverpa armigera* (Insect pest)	Resistant genotypes showed the integrative effect of up-regulated defensive components in leaves, pod walls and seeds such as enhanced activities of catalase, peroxidase, glutathione reductase. Polyphenol oxidase and phenylalanine ammonia lyase, and accumulation of H_2_O_2_ and total phenols	Kaur et al. (2017)
3	Castor	*Fusarium oxysporum* f. sp. *ricini*	Thickening of the cell wall, Increased activities of defense enzymes viz, superoxide dismutase (SOD), peroxidase (POX), catalase (CAT), ascorbate peroxidase (APX), glutathione reductase (GR) and β-1, 3-glucanase (PR protein) in resistant cultivars as compared to the susceptible cultivar	Bharathi et al, (2019)
4	Barley	*Puccinia striiformis* f. sp. *Hordei* (causing stripe rust in barley)	RD2901 (resistant behavior) depicted increased levels of PR proteins, phenylalanine ammonia lyase (PAL), tyrosine ammonia lyase (TAL) and accumulated β-glucan and lignin in the plant cell wall during plant-pathogen interaction	Singla et al. (2020)
5	Rice	*Magnaporthe* *oryzae*	Activity of phenylalanine ammonia lyase (PAL) was induced in rice plants in response to the fungal pathogen	Giberti et al. (2012)
			Bayogenin 3-O-cellobioside accumulated	Norvienyeku et al. (2020)
			Probenazole-inducible protein 1 (PBZ1), and phenylpropanoid accumulated and provide blast resistance	Ma et al. (2020)
6	Wheat	*Alternaria triticina*	Total phenol contents were significantly higher in resistant varieties compared to the susceptible ones	Mishra et al. (2011)
7	Tomato	*Ralstonia solanacearum*	Activities of phenylalanine ammonia lyase (PAL) and polyphenol oxidase were significantly higher in resistant cultivars along with the increase in total phenolic content as compared to the susceptible cultivars	Vanitha et al., (2009)
		*Alternaria alternata*	Chitinase and β-1,3-glucanase induction in tomato cause fruit defense mechanism against *A. alternata* infection	Cota et al. (2007)
8	Arabidopsis	*Powdery mildew*	Elevated early callose deposition results in complete penetration resistance	Ellinger et al. (2013)
		*Pseudomonas syringae*	Glycosylation and accumulation of N-hydroxy pipecolic acid provide defense against *Pseudomonas syringae*	Holmes et al. (2021)
9	*Eruca sativa*	*Alternaria* *brassicicola*	Induction of β-1,3- glucanase and chitinase activities (PR proteins) in the resistant cultivars	Gupta et al. (2013)
10	Stylo	*Colletotrichum gloeosporioides*	Metabolomics showed the increased accumulation of flavonoid compounds and cope with *C. gloeosporioides*	Jiang et al. (2021)

#### Role of carotenoids as non-enzymatic antioxidants

Carotenoids are yellow, red and orange color pigments. C_40_ isoprenoids possess a long-conjugated polyene chain that is accountable for their coloration and biological activities. The unique characteristic of carotenoids, i.e., a polyene backbone together with a chain of conjugated C = C bonds is responsible for both their pigmentation properties and ability to interact with free radicals and singlet oxygen. This property makes carotenoids powerful antioxidants in the plant system (Paznocht et al. 2017). Carotenoids show their anti-oxidative potential by safeguarding the photosynthetic system in the following manners (i) it reacts with the products of lipid peroxidation to end the chain reactions, (ii) scavenges ^1^O_2_ and heat dissipation, (iii) prevents ^1^O_2_ formation by reacting with ^3^Chl^∗^ and excited chlorophyll (Chl^∗^) (Young and Lowe 2018). According to the studies of Mohamad and Bahman (2018), potato plants challenged with *Rhizoctonia solani* and treated with SA showed an increase in carotenoid content which further provided strong antioxidants to the potato plant system resulting in lower disease incidence.

#### Polyamines and their response towards biotic stresses

Polyamines alter in plant cells when they interact with fungal, bacterial and viral pathogens (Asthir et al. 2004) e.g., spermine (Spm), performs a vital function as a mediator in providing defense against the various pathogens thus providing resistance to plants (Takahashi et al. 2004). The accumulation of spermidine in barley leaves was observed after infection with *Puccinia hordei* and Bgh(powdery mildew pathogen). However, it is difficult to investigate the contribution of polyamines in both host plants as well as in pathogenic fungi against stress. But studies exist that indicate the possibilities of controlling fungal diseases in plants by specifically inhibiting the biosynthesis of polyamines (Hussain et al. 2011). Polyamines role in host–pathogen interaction revealed that accumulation of hydrogen peroxide because of spermidine induced degradation of polyamines and nitric oxide served a vital function in the interaction between host plant and pathogens (Yamasaki and Cohen 2006; Di Martino et al. 2013). Hence, manipulation of the key genes or upstream of the polyamine-biosynthesis pathway can impart tolerance against pathogens in plants.

#### Role of phenolics in defense mechanism

Phenols guard the plant against pathogen attack or ultraviolet radiation (Shahidi and Yeo 2016). Phenols can interfere with the oxidation process by reacting with free radicals, chelating-metal ions and by scavenging oxygen (Masisi et al. 2016). As antioxidants, phenolic compounds prevent oxidative damage to cellular organelles and organic molecules such as proteins, membrane lipids, DNA and RNA. Additionally, they function as reducing agents, hydrogen donors and singlet oxygen quenchers (Wang et al. 2018). When pathogens attack, phenolic compounds are produced which are considered as a part of the active defense response in plants (Holub et al. 2019). Cherif et al. (1992) reported that the early and rapid phenolic accumulation at the site of injection results in isolation and limits the progression of pathogens.

Accumulating phenolics possess low molecular weight i.e. benzoic acid and the phenylpropanoids in reaction to infection and leads to slower growth of *B. sorokiniana* in barley and activation of various phytoalexins (Bashyal et al. 2011). Barley possesses higher antioxidant activity than other cereals which are contributed by higher contents of phenolic acids like trans-cinnamic, salicylic, ferulic, chlorogenic, p-hydroxybenzoic, protocatechuic, coumaric and vanillic acids. The different resistant wheat varieties possessed significantly higher total phenol contents as compared to those susceptible to *Alternaria triticina* (Mishra et al. 2011). The activity of PAL expression and the accumulation of phenolic compounds at the infection site has been linked to the resistance mechanism (Nicholson and Hammerschmidt, 1992). In wheat, the level of total phenolic content has been correlated with host resistance to numerous diseases e.g., Karnal bunt (Gogoi et al., 2001) and *Alternaria* blight (Mishra et al. 2011). Metabolomics studies revealed the involvement of phenolic compounds in plant-pathogen interactions (Castro-Moretti et al. 2020; Lopez-Fernandez et al*.* 2020). Phytohormone SA is the most studied defense-responsive phenolic compound (Lefevere et al. 2020). A comparative metabolomic study showed that the accumulation of N–OH-Pip (N-hydroxypipecolic acid) after bacterial infection, imparts SAR (Chen et al*.* 2020).

#### Activation of pathogenesis-related (PR) proteins

In plants, PR proteins are produced to retaliate against various diseases such as fungal, bacterial, viral and viroid diseases, as well as some chemicals. PR proteins were first recognized in tobacco plants infected by the *tobacco mosaic virus* (TMV) (Dani et al. 2005). Most PR proteins exist within the intercellular areas, while, primary PR proteins occur inside the vacuole (Arabi et al. 2019). Some PR proteins are basic and sensitive to degradation by proteolytic enzymes (in the case of tomato and potato). Carbohydrates aid in the synthesis of several defense biomolecules like phenolics and phytoalexins. The metabolism of sucrose, a major translocator of carbon in plants, gets seriously affected during disease (Kosova et al. 2014). Hence, the quality and quantity maintenance of sugars (as well as PR proteins generation and accumulation) against invading pathogens is pivotal.

PR proteins accumulate locally inside the infected and surrounding uninfected tissues hence, restricting the spread of infection to infected parts only. Liu et al. (2010) classified PR-proteins into 17 families like β-1, 3-glucanases, chitinases, peroxidases, thaumatin-like proteins, ribosome-inactivating proteins, thionins, non-specific lipid switch proteins, oxalate oxidase and oxalate oxidase-like proteins. Chitinases, a PR-1 family protein cleaves the bond between C1 and C4 of chitin’s consecutive N-acetylglucosamine (NAG) monomers. Plant chitinases are usually endo-chitinases having the ability to degrade chitin (Suarez et al. 2001). Extracellular chitinases quickly block the spreading of the hyphae that invade internal areas. It also helps to release fungal elicitors that induce the synthesis of several other chitinases inside the host (Stangarlin and Pascholati 2000). Unlike other plant proteins, PR proteins are stabilized through disulfide linkages and hence are resistant to proteolysis and increased temperatures (Gorjanovic 2009). Here we have elaborated the functions of the chitinase and glucanase PR proteins family in disease-resistance.

#### Role of chitinase and glucanase enzymes during pathogen infection

In plants, *β*-1, 3-glucanases belong to the PR-2 family of PR proteins (Ji et al. 2000). *β*-1, 3-glucanases can cleave the *β*-1, 3-glycosidic bond in *β*-1, 3-glucan, a major cell wall component of Oomycetes. In contrast to chitinases, *β*-1, 3-glucanase (called callose in plants) is more important in plant life as indicated from their role in various other physiological functions, apart from plant defense (Anguelova et al. 2001). *β*-1, 3-glucanase plays direct as well as indirect actions to safeguard the plants from fungal pathogens via causing hydrolysis and lysis of fungal cell walls and oligosaccharide elicitors formation for the generation of various PR proteins or phytoalexins, respectively (Ebrahim et al. 2011). Chitinases and *β*-1, 3-glucanase are the most significant hydrolytic enzymes amongst PR proteins produced in several plant taxa after encountering various pathogen infections (Sels et al. 2008), for example, their increased concentration offers protection to the plants against fungal pathogens via degradation of the cell wall as it contains the vital substances i.e., chitin and *β*-1, 3-glucan (Santen et al. 2005). After fungal infection, *β*-1, 3-glucanases expressed in coordination with chitinases as cited from different crops like the bean, pea, tomato, maize, tobacco, soybean, wheat, barley and potato (Sels et al. 2008). Wheat infection with stripe rust fungus (*P. striiformis* f. sp. *tritici*) has increased chitinase activity by upregulating TaBZR2, which confer broad-spectrum resistance (Bai et al. 2021).

#### Transcription factors and regulatory elements

Transcription factors (TFs) regulate gene expression through binding to specific sequences in the promoters of their target genes. TFs play roles at several levels of resistance by transcriptional reprogramming: (a) activate receptor proteins directly by TF (b) basal resistance components expression (e.g., response suppression proteins, receptors, kinases), (c) activation of downstream of receptor initiation (i.e., MAPK cascade leading to TF activation via phosphorylation) (Franco-Zorrilla et al*.* 2014; Lu et al*.* 2011). Upon infection to pathogens, plants respond via altered transcriptional reprogramming of the TF families like AP2/ERF, bHLH, bZIP, MYB, NAC and WRKY (Samota et al. 2017; Tsuda and Somssich 2015). For eg. in rice, Ideal Plant Architecture 1 (IPA1) TF imparts the resistance against rice blast infection by regulating (activating) the expression of WRKY45, a pathogen defense gene (Wang et al. 2018). Similarly, OsWRKY53 imparts resistance against rice blast in rice (Chujo et al. 2007). This indicates WRKY53 plays a prominent role in regulating the release of ROS, throughout the hypersensitive response. TF TaRIM1 (*R. cerealis*-induced MYB confers resistance to sharp eyespot disease in wheat via modulating defense genes (Shan et al. 2016). Similarly, ORK10/LRK10 are defense regulator receptor kinases that impart resistance against fungal diseases in cereal crops (Marcel et al*.* 2010).

The regulatory element, GCC-box elements represent the hallmark of the promoters of aphid- and pathogen-responsive genes (Dong et al. 2010). Conclusively, defense genes such as *WRKY53* impart broad-spectrum resistance through the transfer of wide-stream responsive signals to the other defense-related genes in proximity (upstream and downstream). Hence, the signaling crosstalk of the TFs for disease resistance seems a promising domain of research in crops.

## How can plant metabolites be used in crop improvement?

Metabolomics measures the metabolite abundance and environmentally induced changes in metabolites concentration, as a predictive biomarker for disease diagnosis. Metabolic markers are a sub-category of biomarkers, which reflect the compounds involved in plant metabolism (Fernandez et al. 2016; Zaynab et al. 2019). During biotic stresses, plants accumulate numerous metabolites often tissue and species-specific, that can function as biomarkers for biotic stress resistance (Razzaq et al. 2019). Such metabolites that can be used to provide tolerance against biotic or abiotic stresses are regarded as defensive diagnostic or metabolite markers. In plants, single metabolic markers have been suggested to assess the stress intensity, e.g., proline, which accumulates drought stress-prone plant species (Hayat et al. 2012). Later, metabolic variables were found to be useful markers to detect stress damage or resistance and hence the diagnostic markers have been developed in the form of enzymes (Gibon et al. 2004), metabolites (Korn et al. 2010; Duan et al. 2021), transcripts (Tamaoki et al. 2013) and amino acids (Zhao et al. 2021).

### Metabolic markers for disease resistance in crops

Fridman et al. (2000) first proposed metabolic markers in 2000, as a tool to map the metabolite quantitative trait loci (mQTLs) and find the related candidate genes. In 2007 for the very first time, metabolic profiling was carried out to evaluate the biomass performance in *Arabidopsis thaliana (*with a coefficient of correlation of 0.58) that opened new avenues in plant breeding where metabolic markers can be explored to find the allelic combinations for better plant performance (Meyer et al. 2007). Hence, metabolomics became an emerging technique for studying plant immunity, especially in interpreting the functions of small-sized molecules associated with plant—microbe interactions (Feussner and Polle 2015). Phytoalexins and pathogenesis-related proteins are potential metabolite markers against wilt pathogen, *Fusarium oxysporum* in chickpea roots (Kumar et al. 2016). Similarly, Desalegn et al. (2016) identified the function of pisatin and pisatin biosynthesis-associated proteins in conferring resistance against *Didymellapinodes *in *Pisum sativum*. Lately, Seybold et al. (2020) performed metabolomics in wheat to elucidate the resistance mechanism against *Z. tritici* causing disease-resistant metabolites, i.e., trehalose, asparagine, phenylalanine, myoinositol, and L-alanine have been reported to serve as unique metabolite markers against *Fusarium graminearum* causing head blight in wheat (Cuperlovic-Culf et al. 2018). Zhou et al. (2019) reported smiglaside and smilaside as potential biomarkers against the *Fusarium graminearum* (head blight) in maize. Khizar et al. (2020) identified metabolites like phenylpropanoids (stilbenes and furanocoumarin), flavonoids (phlorizin and kaempferol), alkaloids (indolizine and acetylcorynoline) and terpenoids (azelaic acid and oleanolic acid) for leaf spot resistance (*Aspergillus tubingensis*) in cotton. Lately, Zhao et al. (2021) identified proline and alanine as important metabolic markers for head blight resistance in wheat. In a recent study by Duan et al. (2021), metabolites namely shikimate, galactinol, trehalose, D-mannose, linolenic acid, dopamine, tyramine, and L-glutamine were identified as metabolic markers imparting plant defense response against rice blast. The list of different plant metabolic markers identified to provide resistance against the diseases has been given in Table 4.

**Table 4:** Different metabolic markers associated with plants to impart resistance against stress

Plants	Resistant against	Statistical approach	Metabolite accumulated/Reduced	References
Wheat	*Fusarium* *graminearum*	Fold change/ Correlation Network	Phenolic acid, Phenylpropanoids,Trehalose, Asparagine, Phenylalanine, Myoinositol,3-hydroxybutarate, and L-alanine, Spermine, Putrescine, GABA, Inositols, Galactose, and Lactic acid	Cuperlovic-Culf et al. (2016, 2018) Gunnaiah et al. (2012)
	Wheat streak mosaic virus	PCA, KEGG, METLIN, MetFrag and MetaboAnalyst	Reduction in some amino acids such as L-tyrosine, tryptophan, isoleucine and phenylalanine	Farahbakhsh et al. (2019)
	*Triticum* *turgidum*	PCA, XCMS and CAMERA	Benzoxazinoids	Shavit et al. (2018)
	*Fusarium graminearum*	PLS-DA	Proline and Alanine	Zhou et al*.* (2021)
Rice	*Rhizoctonia* *solani*	-	Jasmonic acid, mucic acid, and glyceric acid	Suharti et al. (2016)
	Xanthomonas oryzaepv. *oryzae*	KEGG, MassHunter, GeneSpring-MS 1.2 and METLIN	Phenylalanine and glutamine, linoleic acid lipids, carbohydrates, alkaloids, xanthophylls, and acetophenone	Sana et al. (2010)
	*Orseolia oryzae*	ANOVA	Heneicosanoic acid, Threonic acid, Palmitoleic acid, Palmitic acid, Nonadecanoic acid and Linoleic acid	Agarrwal et al. (2014)
	Magnaporthe oryzae	PCA, partial least squares discriminant analysis (PLS-DA), and orthogonal partial least squares discriminant analysis (OPLS-DA)	Shikimate, galactinol, trehalose, D-mannose, linolenic acid, dopamine, tyramine, and L-glutamine	Duan et al. (2021)
Maize	*Fusarium graminearum*	ANOVA and SAS software	Smiglaside and Smilaside	Zhou et al, 2019
Cotton	Aspergillus tubingensis	PCA, OPLS-DA, PLS-DA	Phenylpropanoids (stilbenes and furanocoumarin), flavonoids (phlorizin and kaempferol), alkaloids (indolizine and acetylcorynoline) and terpenoids (azelaic acid and oleanolic acid)	Khizar et al. (2020)

### Defense metabolites targeted breeding approaches

Metabolomics based genome-wide association studies (mGWAS) and metabolic QTLs (mQTLs) are powerful and potential tools in detecting genetic variations associated with different metabolites in plants. Metabolic profiling helps in refining the genotype–phenotype association through the investigation of different important metabolites that provide biotic stress resistance. The mQTL/mGWAS helps in the identification of SNP markers-metabolites association resulting in pointing out the candidate genes governing biotic stress tolerance (Fernandez et al. 2016; Wen et al. 2016). However, the profiling of metabolites using electrospray ionization tandem mass spectrometry in mapping or GWAS (the sample size is quite large) is very costly and hence, is expensive to apply for a large sample size (Gieger et al. 2008; Gibon et al. 2012). Therefore, to study the genotype-metabolite associations, the bulk-sequencing-based approach like QTL-Seq can be a relatively more effective approach. In the bulk-seq approach, the bulk of around 15–10 extreme phenotypes-resistant and susceptible (based on plant-pathogen reaction) can be selected and used for the metabolomics (Zou et al. 2016). Another approach is to use the subpanels of the germplasm but by ensuring the presence of diversity in sub-panels as the true indicator of the whole germplasm. Such approaches allow identifying a small set of reliable metabolite markers (hence reducing the cost) which can be employed by plant breeders for the effective selection of tolerant germplasm. Later, identification of key genetic markers related to such metabolomics markers can further help to reduce the cost of metabolomics-based breeding programs as sequencing-based markers are cheaper as compared to metabolite markers. Further, forward and reverse genetics can help validate the identified candidate genes for their role in the targeted metabolites synthesis.

**Fig. 4:**
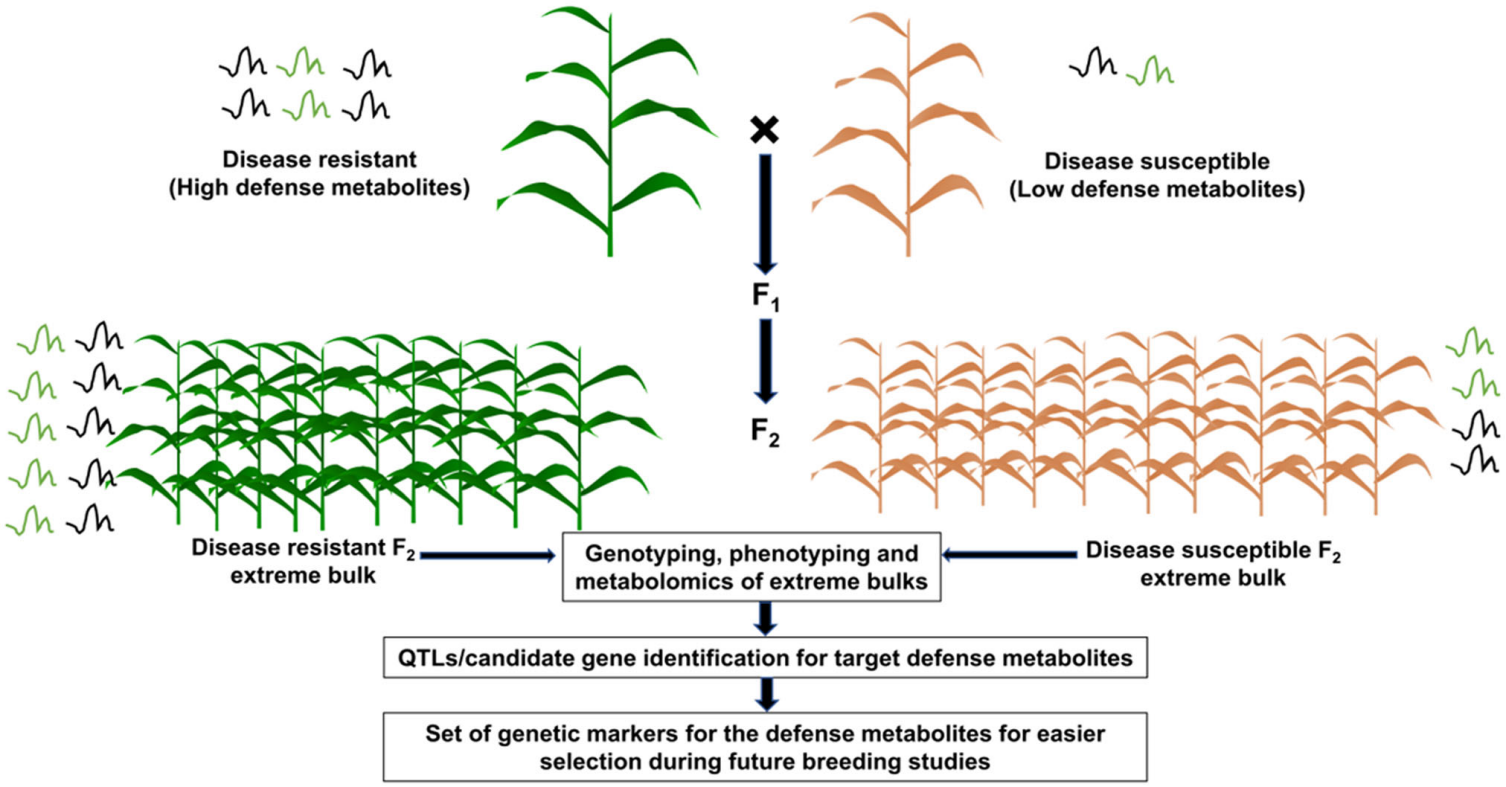
Genomics assisted breeding approach for utilization of defense metabolites. The F_2_ population is generated by crossing the two contrasting genotypes and the extreme bulks identified on screening under artificial inoculation conditions (disease resistant and susceptible) are subjected to whole genome sequencing and metabolomics. One of the parents genome sequence is used as a reference to identify putative candidate genes and associated SNPs. The identified SNPs can serve as effective markers for the selection of resistant genotypes under future breeding programs such as marker assisted selection. Similarly, mGWAS concept can be applied to a representative diverse set of germplasm and establish marker-trait associations

The greater use of metabolites in crop improvement has been witnessed due to two important reasons. First, is the development of advanced next-generation sequencing platforms that provided ultra-high-density maps for the identification of mQTLs/candidate genes (Scossa et al. 2015). The second reason is the development and availability of easy-to-use, open-source and efficient statistical platforms for fast-forward analysis of metabolic and phenotypic data. For example, MetabR (http://metabr.r-forge.r-project.org/), MetaboAnalystR(https://github.com/xialab/MetaboAnalystR), and MetaboDi (http://github.com/ and reasmock/MetaboDi/a) are preferred R software packages for analysis of metabolomics due to open-source nature. The metabolomics can be integrated with the genomic sequencing technologies-based approaches (like transcriptomics and pan-genomics) to exploit the metabolic diversity in plants (Zhou and Liu 2022). The process of genomics assisted breeding approach for utilization of defense metabolites is represented in Fig. 4

## Concluding remarks and future perspectives

The optimum and sustainable strategy for the management of plant diseases should be the targeted manipulation of naturally occurring defense mechanisms of host plants. In the recent past, remarkable efforts have been made for the identification of metabolites providing disease resistance in different crops. Different metabolites like enzymes, amino acids, lipids and organic acids have been identified as metabolic markers for disease resistance in different crops. Hence, the target of disease breeding programs should focus on the identification of metabolite markers involved in providing the resistance. The metabolic markers being more realistic performance indicator of the plants can be more effective than the molecular markers. However, the use of metabolite markers is a costly affair, especially when dealing with larger germplasm. Hence, such established metabolite markers can further be used to identify the closely linked genetic markers for diseases. The identified markers can be further validated and used for the identification of mQTLs and mGWAS. Such metabolites can also be used for genome-wide prediction to enhance the genetic gains in disease resistance breeding. Furthermore, the use of metabolites can be extended to epigenome-wide association studies and pan-genomics. The availability of the phenotypic data on the genotype’s performance from the high throughput phenomics approach would also add an additional layer to correlate and establish relationships with the different metabolites. Hence, the collaborations between plant breeders, pathologists, statisticians and bioinformaticians would prove crucial to explore the omics (genomics, phenomics, metabolomics) to their fullest potential. Recently, the emerging approach of metabolome network studies (interactions between the different metabolites) opens the avenue for exploring the interaction of different metabolites in imparting disease. The utilization of resistance-related constitutive and induced metabolites in imparting plant disease resistance would be an interesting research domain to explore in near future. Therefore, it’s the need of the hour to explore the natural metabolites in host plants to manage plant diseases effectively and sustainably.
